# Inhibition of HIF1α-Dependent Upregulation of Phospho-l-Plastin Resensitizes Multiple Myeloma Cells to Frontline Therapy

**DOI:** 10.3390/ijms19061551

**Published:** 2018-05-23

**Authors:** Manon Bosseler, Vanessa Marani, Angelina Broukou, Amandine Lequeux, Tony Kaoma, Vincent Schlesser, Jean-Hugues François, Valérie Palissot, Guy J. Berchem, Nasséra Aouali, Bassam Janji

**Affiliations:** 1 Laboratory of Experimental Cancer Research, Luxembourg Institute of Health (LIH), L-1526 Luxembourg City, Luxembourg; manon.bosseler@lih.lu (M.B.); vanessa.marani@lih.lu (V.M.); a.broukou@gmail.com (A.B.); amandine_lequeux@msn.com (A.L.); Berchem.Guy@chl.lu (G.J.B.); nassera.aouali@lih.lu (N.A.); 2 Bioinformatics and Modelling, Luxembourg Institute of Health (LIH), L-1526 Luxembourg City, Luxembourg; tony.kaoma@lih.lu; 3 Laboratory of Hematology, Centre Hospitalier de Luxembourg (CHL), L-1526 Luxembourg City, Luxembourg; Schlesser.Vincent@chl.lu (V.S.); francois.jean-hugues@chl.lu (J.-H.F.); 4 Laboratory of Oncolytic-Virus-Immuno-Therapeutics, Luxembourg Institute of Health (LIH), L-1526 Luxembourg City, Luxembourg; Valerie.palissot@lih.lu

**Keywords:** drug resistance, MM, IMiDs, PIs, HIF1α, l-Plastin

## Abstract

The introduction of novel frontline agents in multiple myeloma (MM), like immunomodulatory drugs and proteasome inhibitors, has improved the overall survival of patients. Yet, MM is still not curable, and drug resistance (DR) remains the main challenge. To improve the understanding of DR in MM, we established a resistant cell line (MOLP8/R). The exploration of DR mechanisms yielded an overexpression of HIF1α, due to impaired proteasome activity of MOLP8/R. We show that MOLP8/R, like other tumor cells, overexpressing HIF1α, have an increased resistance to the immune system. By exploring the main target genes regulated by HIF1α, we could not show an overexpression of these targets in MOLP8/R. We, however, show that MOLP8/R cells display a very high overexpression of *LCP1* gene (l-Plastin) controlled by HIF1α, and that this overexpression also exists in MM patient samples. The l-Plastin activity is controlled by its phosphorylation in Ser5. We further show that the inhibition of l-Plastin phosphorylation restores the sensitivity of MOLP8/R to immunomodulatory drugs (IMiDs) and proteasome inhibitors (PIs). Our results reveal a new target gene of DR, controlled by HIF1α.

## 1. Introduction

Multiple myeloma (MM) is a malignant disorder involving uncontrolled proliferation of plasma cells within the bone marrow [1]. Last two decade, the therapeutic strategy in the fighting of MM has greatly changed, improving quality of life and survival. Yet, MM is still an incurable cancer [2], due to the development of drug resistance [3,4].

The drug resistance can emanate from several genetic abnormalities observed in MM patients [5]. Some of these genomic instabilities have been related to drug resistance and relapse of the disease, such as the translocation t(4; 16) and t(14; 20), demonstrated by Greco et al. [5,6]. The serial drug treatment followed by relapses in MM patients cause a drug resistance phenotype, called “acquired resistance” [7]. The acquired resistance involves several pathways [8,9], of which the most investigated is the overexpression of ATP-binding cassette (ABC) transporter family, including P-glycoprotein (Pgp), multidrug related protein-1 (MPR1), and breast cancer resistance protein (BCRP) [10]. Pgp is the he main protein involved in multidrug resistance (MDR) in MM [10,11]. Pgp is a pump preventing the drug to reach its target by expulsing it out of the tumor cells [12]. The role of ABC transporter in the resistance to chemotherapy is well studied [10,13], and to overcome the activity of these proteins, the best strategy is to inhibit them by molecules.

For decades, the strategy to fight the disease has been completely modified. In fact, the development of MM is localized in the bone marrow, constituting the microenvironment which participates intimately in the progression of the disease, cell proliferation, angiogenesis, metastasis, and drug resistance [7,14,15]. The bone marrow microenvironment is a very complex structure composed of different cell types belonging to the innate and adaptive immune systems, hematopoietic stem cells, and growth factors and cytokines [14,16]. Recently, another feature of the microenvironment which has a crucial impact in cancer development has been reported, namely the low oxygen tension (hypoxia) area [17,18]. The adaptation of tumor cells to the hypoxic condition is regulated by the hypoxia inducible factor-1α (HIF1α) [19,20,21]. In order to adapt to hypoxia, HIF1α activates target genes having a hypoxia responsive element (HRE) upstream of their promoters [22]. These HIF1α-regulated genes promote tumor progression, metastasis, and drug resistance [23,24,25].

The hypoxic condition in MM promotes the expansion of malignant plasma cells in other areas of the bone marrow. In order to spread out, malignant plasma cells have to modify the organization of the cytoskeleton [26]. The dynamic of the cytoskeleton is under the control of the actin binding protein l-Plastin [27,28]. The activity of l-Plastin is regulated by its phosphorylation on Ser5 [28,29], and this Phospho l-Plastin is involved in the adhesive function of tumor cells and in tissue invasion.

The main drugs used in the frontline treatment of MM, and in the consolidation and maintenance of the therapy, are immunomodulatory drugs (IMiDs) and/or proteasome inhibitors (PIs), which are known to act on the microenvironment, as well as on the tumor cells. In fact, it has been demonstrated that drugs, such as bortezomib (proteasome inhibitor) prevent the mechanism underlying HIF1α [30]. In addition, Lu et al. demonstrated that lenalidomide (IMiD) impedes angiogenesis and metastasis in normoxic and hypoxic conditions [31,32]. Nevertheless, drug resistance is still the main obstacle to overcome in MM.

In this work, we set up a resistant cell line to doxorubicin (DOX) from MM cell line, MOLP8. We demonstrate that this cell line is resistant to pomalidomide and carfilzomib, the latest IMiD and PI validated by the FDA (Food and Drugs Administration), but it is sensitive to dexamethasone, whereas the MOLP8 cell line is resistant to this drug. The resistant cell line, which overexpresses Pgp, shows also a strong expression of HIF1α, although the cell line was set up in normoxic conditions. The study of the degradation of HIF1α in normoxia has been investigated in detail. Our data show that the pathway involved in the degradation of HIF1α in normoxia is impaired. Moreover, in the MOLP8/R cell line, the overexpression of HIF1α confers a resistance to the microenvironment of our cell line. The study highlights a new HIF1α-regulated gene in MOLP8/R cells, namely LCP1. This protein, l-Plastin, activated by its phosphorylation in Ser5, is also overexpressed in our resistant clone. We demonstrate that the inhibition of l-Plastin phosphorylation leads to sensitivity of MOLP8/R to PIs and Pomalidomide.

In this study, we thus identified a new protein regulated by HIF1α, and involved in MDR of MM, which can be a potential target to overcome drug resistance in MM.

## 2. Results

### 2.1. Drug Resistance Study to New Drugs Used in MM Treatment

**Figure 1:**
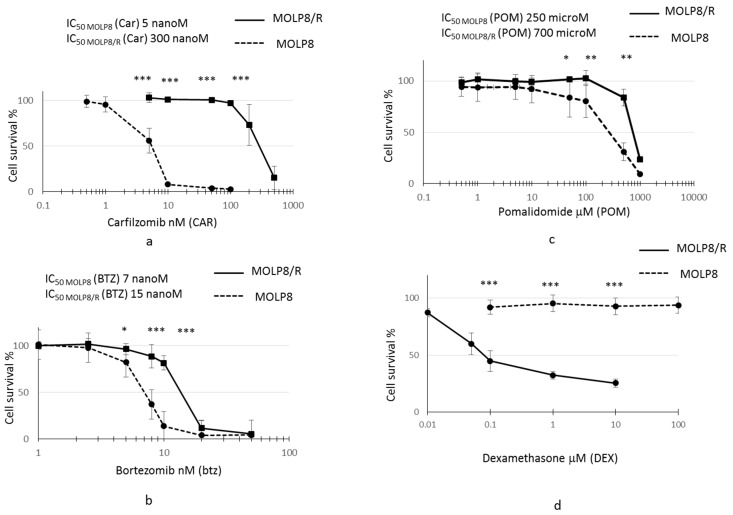
Study of drug resistance of MOLP8/R cell line to immunomodulatory drugs (IMiDs) and proteasome inhibitors (PIs). Both cell line MOLP8 and MOLP8/R are treated for 48 h with the different molecules used in the multiple myeloma (MM) treatment, carfilzomib (Car), bortezomib (Btz), pomalidomide (POM), and dexamethasone (DEX). MOLP8 curve is represented by black dotted line and MOLP8/R curve is represented by a black full line. MOLP8/R cell line is resistant to PIs with resistance index (IR) (IC_50_ MOLP8/R/ IC_50_ MOLP8) 60 for Car (**a**); IR to Btz 2.14 (**b**). In addition, MOLP8/R cell line is resistant to the IMiD tested, POM, with an IR of 2.8 (**c**), whereas the MOLP8/R cell line is sensitive to DEX (**d**). * *p* < 0.05, ** *p* < 0.01 and *** *p* < 0.001.

After selection of the resistant clone from MOLP8 cell line with DOX, we investigated the sensitivity of MOLP8/R cell line to PIs (bortezomib and carfilzomib) (Figure 1a,b) and IMiD, Pomalidomide (Figure 1c), and also to dexamethasone (Figure 1d). The resistant clone MOLP8/R is 60 and 2.15 times more resistant to carfilzomib and bortezomib, respectively, than the MOLP8 cell line. Moreover, the MOLP8/R cell line is also 3 times more resistant to pomalidomide than MOLP8. Surprisingly, the MOLP8/R cell line is much more sensitive to dexamethasone, compared to MOLP8 cells (Figure 1d).

### 2.2. Measure of Proteasome Activity

**Figure 2:**
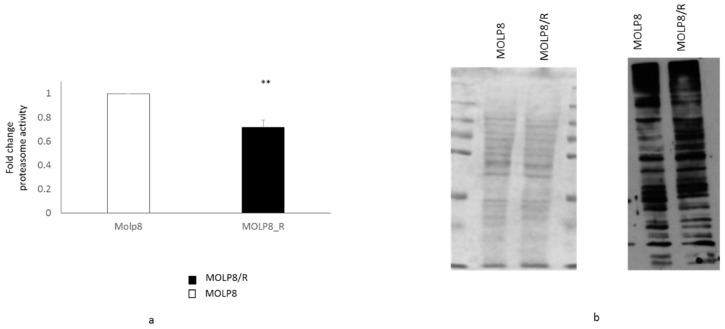
Study of proteasome activity in sensitive and resistance cell line. Measure of proteasome subunit 20S activity of the proteasome in MOLP8 (white) and MOLP8/R cell line (black histogram) (**a**) by using the kit Amplite Fluorimetric Proteasome 20S activity assay. Determination of the ubiquitination protein quantity in MOLP8 and MOLP8/R cell lines by Western blot (**b**). The left panel represents the PVDF membrane stained with the Ponceau Red, showing that the quantity of protein loading is the same for both cell lines. The right panel represents the photographic film resulting from the incubation of the membrane with ubiquitinated antibody overnight. ** *p* < 0.01.

We have studied the proteasome activity in MOLP8/R and MOLP8 cells by measuring the chymotrypsin-like protease activity associated with the proteasome complex (Figure 2a). The result shows that the proteasome activity in MOLP8/R cell line is significantly lower than in MOLP8 cells. We further explored the level of ubiquitinated proteins in both cell lines MOLP8 and MOLP8/R by Western blot, using an ubiquitinated antibody (Figure 2b). The level of ubiquitinated protein in MOLP8/R cell line is higher than in MOLP8 cell line (Figure 2b right). This result indicates that there was an accumulation of ubiquitinated proteins in the resistant cell line, indicating an impaired proteasome function. The quantity of loaded proteins is the same for both cell lines (Figure 2b left).

### 2.3. Overexpression of HIF1α, HIF2α, and HIF-OH in MOLP8/R Cell Line

**Figure 3:**
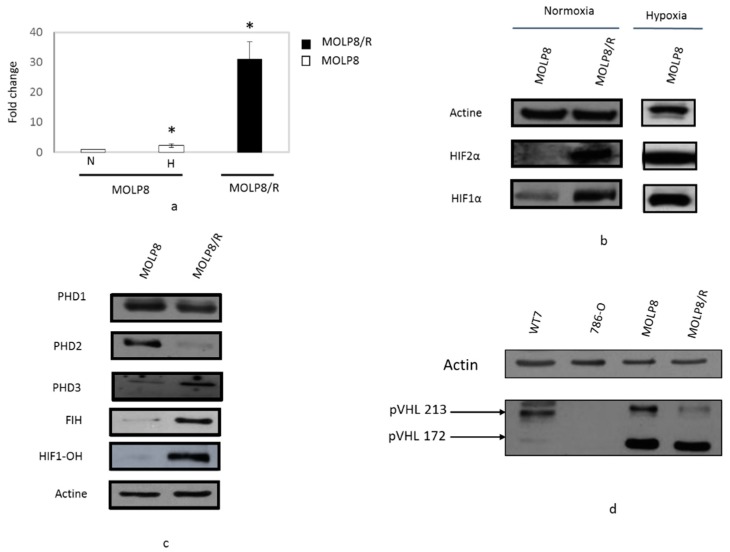
Study of the HIF network regulated in normoxic conditions. Comparative expression of HIF2α by q-PCR (**a**) and by Western blot (**b**) in MOLP8 and MOLP8/R cell line in normoxic condition, as a positive control MOLP8 cells were incubated 24 h of hypoxia. Study of protein expression involved in the HIF1α degradation in normoxia by western blot, proline hydroxylases and asparagyl hydroxylase HIF (FIH) (**c**) and VHL (**d**). WT7 and 786-O are used, respectively, as a positive and negative control for VHL. * *p* < 0.05.

Whereas mRNA of *HIF2α* gene in MOLP8/R is more than 30 times overexpressed compared to the level of *HIF2α* gene in the MOLP8 cell line under hypoxic condition for 24 h (white box) (Figure 3a). By Western blot, we validated the overexpression of HIF2α in the resistant cell line (Figure 3b). For the investigation of HIF1α expression, as a positive control, MOLP8 cell line was incubated in hypoxic conditions for 24 h, and as expected, under hypoxia, MOLP8 cells have strong expression of HIF1α. In MOLP8/R cells we also found HIF1α expression, also in normoxic condition (Figure 3b). Although HIF1α and HIF2 are overexpressed in MOLP8/R cell line, we focused our work on HIF1α.

### 2.4. Study of Degradation Pathway of HIF1α in Normoxia Conditions

In normoxia, HIF1α is hydroxylated by three enzymes, Proline HyDroxylases (PHD) PHD1, PHD2, and PHD3. Once hydroxylated, HIF1α, now called HIF1-OH, binds to a complex, E3 ubiquitin ligase complex. This big complex is identified by Von Hippel-Lindau tumour suppressor (pVHL or VHL), allowing its ubiquitination, and this big structure is degraded by the proteasome [33].

The expression of two of these proline hydroxylases are deregulated in our resistant clone. Indeed, PHD2 is not expressed in MOLP8/R, whereas PHD3 is overexpressed in MOLP8/R (Figure 3c). We also explored the expression of VHL in both cell lines MOLP8 and MOLP8/R; as a negative control, we used the 786-O cell line, which is mutated in the VHL gene in the wild type cell line, and as a positive control we used WT7 cell line derivating from 786-O cell line stably transfected with pRC-HA-VHL vector [34]. Thus, the Western blot is done with the four different cell lines which showed that the expression of VHL isoform p213 has a much lower expression in MOLP8/R, compared to MOLP8 and WT7 cell (Figure 3d).

HIF1α in normoxia can be hydroxylated by another pathway that involves the enzyme asparagyl hydroxylase (FIH). By examining its expression by Western blot, we show that in MOLP8/R, we have strong expression of FIH compared to the sensitive cell line. In addition, the study of the expression of HIF-OH in MOLP8 and MOLP8/R reveals also its overexpression in MOLP8/R (Figure 3c).

### 2.5. Resistance to NK Cell Lysis

**Figure 4:**
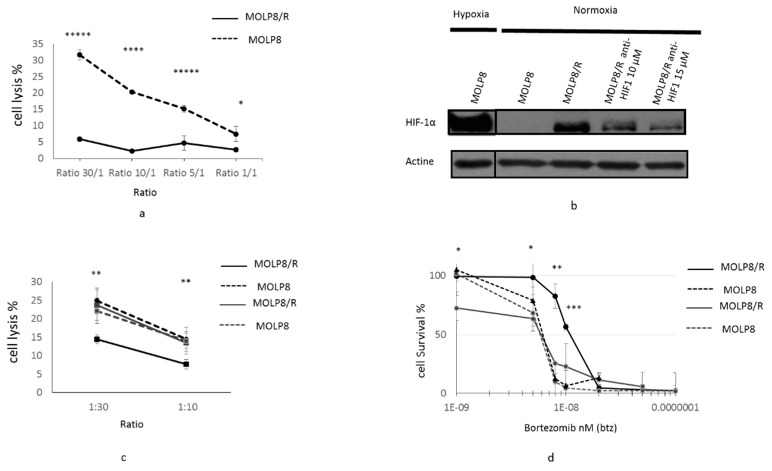
Resistance of MOLP8/R cell line to the immune system: role of HIF1α. Tumor cell lysis MOLP8 and MOLP8/R by NK92MI cell line at different ratios (tumor cells/NK cells). Tumor cells are incubated for 4 h in contact with NK cells, then stained with carboxyfluorescein succinimidyl ester (CFSE) and Thiazol Orange PRO (TOPRO). The double positivity of tumour cells by both dyes are determined by flow cytometry. Black dotted line is MOLP8, and black line is MOLP8/R (**a**). Involvement of HIF 1α in resistance to NK cell lysis. Both cell lines MOLP8 and MOLP8/R are treated for 48 h with an anti-HIF1α drug (cryptotanshinone) at two different concentrations, 10 and 15 µM. The validation of the inhibition of HIF1α in MOLP8/R is verified by Western blot (**b**). Afterwards, treated cell lines MOLP8/R and MOLP8 are incubated for 4 h with NK cells, and the lysis is evaluated by flow cytometry. Black dotted line is MOLP8, and black line is MOLP8/R, grey dotted line is MOLP8 after treatment, and grey line is MOLP8/R (**c**). Involvement of HIF1α in drug resistance to Btz, Molp8, and MOLP8/R is treated and co-treated for 48 h with Btz, or Btz plus cryptotanshinone at 10 µM, and the cytotoxicities are calculated. Black dotted line is MOLP8, and black line is MOLP8/R, and after treatment, grey dotted line is MOLP8, and grey line is MOLP8/R (**d**). * *p* < 0.05, ** *p* < 0.01, *** *p* < 0.001, **** *p* < 0.0001 and ***** *p* < 0.00001.

We investigated the sensitivity of MOLP8 and MOLP8/R cells to cytotoxic lysis by Natural Killer (NK) cells. We used different ratios of tumor/NK cells: 1:1, 1:5, 1:10, and 1:30, (Figure 4a). In conclusion, for all ratios, the MOLP8/R cell line is resistant to NK cell lysis compared to MOLP8 cells (Figure 4a).

In order to verify, the involvement of HIF1α in the resistance of MOLP8/R cell line to NK cell lysis, we treated the tumor cells with HIF1α inhibitor cryptotanshinone at two different concentrations, 10 and 15 µM, for 48 h. First we verified the inhibition of HIF1α expression by Western blot (Figure 4b). As a positive control, we used MOLP8 cells incubated in hypoxic conditions. The immunoblot illustrates a decrease of HIF1α expression at 10 and 15 µM of cryptotanshinone compared to MOLP8/R without treatment in normoxia, and also to MOLP8 in hypoxia (Figure 4b). Then, we considered the NK cell susceptibility of treated MOLP8/R and MOLP8 cell lines with cryptotanshinone to NK cell lysis. After treatment with the cryptotanshinone for 48 h, we restored the sensitivity of MOLP8/R to NK cell lysis at an identical level as MOLP8 cells (black and dots curve) (Figure 4c).

The involvement of HIF1α in the drug resistance to Btz has been then investigated in both cell lines. The co-treatment Btz and cryptotanshinone anti-HIF1α (10 µM) for 48 h on MOLP8/R cells restored also the sensitivity to Btz (Figure 4d). In fact, the light grey curve (MOLP8/R co-treated) and the black dot curve (MOLP8 treated with Btz alone) are overlapping.

### 2.6. Main Reference Genes Targeted by HIF1α Transcription Factor

**Figure 5:**
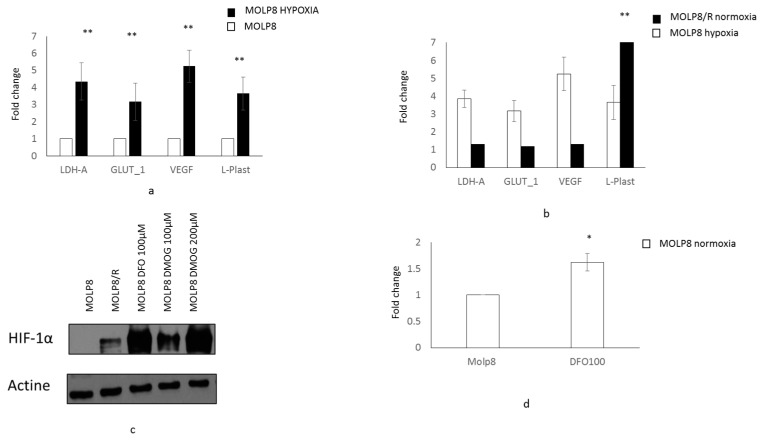
Regulation of l-Plastin by the transcription factor HIF1 α in MOLP8/R. Expression of reference genes (*LDH-A*, *GLUT-1*, and *VEGF*) and *LCP1* gene by q-PCR (l-Plastin) in MOLP8 cell line on normoxic and hypoxic condition for 24 h (no color box MOLP8 in normoxia, MOLP8 in hypoxia black box) (**a**). Expression of reference genes and *LCP1* gene in MOLP8/R cell line in comparison with MOLP8 cell line in hypoxia (**b**). Study of the expression of HIF1α by Western blot in MOLP8/R cell line treated with DFO and DMOG 6 h in normoxia in comparison with MOLP8/R (**c**). *LCP1* gene expression by q-PCR in MOLP8 cell line treated (or not) for 6 h with DFO at 100 µM in normoxia (**d**). * *p* < 0.05, ** *p* < 0.01.

Hypoxia upregulates genes involved in angiogenesis, the vascular endothelial growth factor (*VEGF*), and the adaptation to glucose metabolism, glucose transporter (*GLUT1*), and lactate dehydrogenase A (*LDH-A*). Thus, we studied the mRNA expression of these three genes by q-PCR. As a positive control, MOLP8 cells were exposed to hypoxia for 24 h. We also tested *LCP1* gene (l-Plastin gene), which is strongly overexpressed in the MOLP8/R cell line. We compared the expression of the four genes in the MOLP8/R cell line incubated in normoxia, to MOLP8 cell line exposed for 24 h to hypoxia. Under hypoxia MOLP8 cell line has an upregulation of *VEGF*, *LDHA*, and *GLUT1* genes compared to MOLP8 cells in normoxia. Moreover, we detected an upregulation of *LCP1* gene in MOLP8 cells in hypoxia (Figure 5a). Although HIF1α is overexpressed in MOLP8/R cells in normoxic condition, we did not observe an overexpression of its downstream genes *VEGF*, *LDHA*, and *GLUT1* (Figure 5b). However, we had a very strong overexpression of LCP1 gene in MOLP8/R cells (Figure 5b).

In order to study if there is a link between the expression of l-Plastin and HIF1α, we treated MOLP8 cells with drugs preventing the degradation of HIF1α by prolyl hydroxylase or by asparagyl hydroxylase in normoxic conditions. The expression of HIF1α in MOLP8 cells treated with DesFerriOxamine (DFO) and Dimethyloxalylglycine (DMOG) was validated by Western blot (Figure 5c). MOLP8 cells were treated with DFO at 100 µM for 6 h. After mRNA extraction from the cells, we studied the expression of l-Plastin by q-PCR. The data shows a 1.6-fold increase of LCP1 gene level compared to untreated MOLP8 control cells (Figure 5d).

### 2.7. l-Plastin and Phospho l-Plastin Expression in Normoxia and Hypoxia

**Figure 6:**
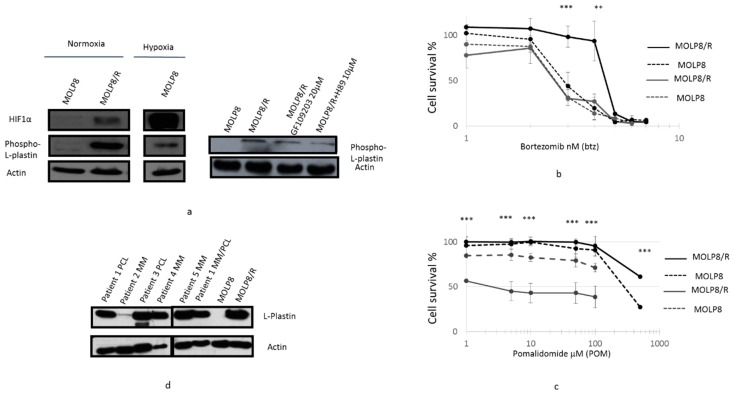
Involvement of Phospho l-Plastin in DR to IMiDs and PIs. Inhibition of Phospho l-Plastin expression in MOLP8/R cell line treated with protein kinase inhibitors. Both cell lines, MOLP8 and MOLP8/R, are treated for 48 h with GF109203 or with H89. After protein extraction from treated cells, the expression of Phospho l-Plastin is investigated by Western blot (**a**). Restoration of cell sensitivity in MOLP8 andMOLP8/R cell line to Btz treated with H89. Both cell lines are cotreated with a range of Btz concentration and 10 µM H89 for 48 h. Black dotted line is MOLP8, and black line is MOLP8/R, after treatment, grey dotted line is MOLP8, and grey line is MOLP8/R (**b**). Effect of Dexamethasone on the cytotoxicity to pomalidomide on MOLP8 and MOLP8/R. Co-treatment of MOLP8 and MOLP8/R cell lines with DEX and a range of POM concentrations for 48 h. Black dotted line is MOLP8, and black line is MOLP8/R, after treatment, grey dotted line is MOLP8, and grey line is MOLP8/R (**c**). Expression of l-Plastin in MM patient samples at different stages of the disease investigated by Western blot (**d**). ** *p* < 0.01 and *** *p* < 0.001.

The study of Phospho l-Plastin expression in both cell lines by Western blot showed a strong overexpression of Phospho l-Plastin in MOLP8/R, compared to the sensitive cell line (Figure 6a left panel). Also in MOLP8 cells incubated in hypoxia, we had an overexpression of Phospho l-Plastin (Figure 6a left panel). Yet, the expression of the protein in MOLP8/R is much stronger than MOLP8 in hypoxia (Figure 6a left).

l-Plastin is phosphorylated at serine 5 [29] by two protein kinases, protein kinase A (PKA) [35] and protein kinase C (PKC) [36]. The treatment of MOLP8/R with two drugs, H-89 and bisindolylmaleimide-1 (Go6793) targeting PKA [37] and PKC, respectively, demonstrated a decrease of phospho-l-plastin expression (Figure 6a right panel). However, the effect of H89 on the decrease of phospho-l-plastin expression is more important than the treatment with (G06793) (Figure 6a right).

In addition, the co-treatment of MOLP8 and MOLP8/R with H89 at 2 µM and a dose range of bortezomib for 48 h restored the sensitivity of MOLP8/R to bortezomib at the same level as the MOLP8 cell line Figure 6b). We noticed that H89 has no effect on the sensitivity to bortezomib of MOLP8 (Figure 6b). In addition, the co-treatment for 48 h of pomalidomide with dexamethasone shows a very strong decrease of cell line viability in MOLP8/R, compared to MOLP8 cell line (Figure 6c).

The investigation of l-Plastin expression in samples of MM and Plasma Cell Leukemia (PCL) patients displayed an overexpression of l-Plastin, in PCL patients and MM/PCL patients, as shown by Western blot (Figure 6d). As a positive and negative control, we used MOLP8 and MOLP8/R cell lines (Figure 6d).

## 3. Discussion

Since the introduction, in MM treatment, of the IMiDs and PIs, that target the microenvironment of tumor cells, the quality of life and survival of MM patients have increased [33,38,39]. Unfortunately, most of patients relapse and develop progressive disease [40], leaving only a restricted choice of treatment for these patients, which have now relapse/refractory MM (RRMM) [41]. In view of the inadequacy of treatment of RRMM patients, new molecules are urgently required. The emergence of antibody therapy, anti-CD38 and anti-SLAMF7, allowed to improve the choice of treatments in relapse and refractory MM patients. Although these agents showed no or low activity as single agents, while daratumumab showed slightly better results, even in monotherapy [42]. However in association, however, these drugs gave very promising results in phase 2 clinical trials, like the SIRIUS trial [43], and in the phase 3 CASTOR [44] and POLUX trials done with daratumumab [45] or ELOQUENT-2, done with elotuzumab [46]. These trials, were realized with the antibody either alone or in combination with bortezomib/dexamethasone or lenalidomide/dexamethasone [47,48]. Thus, in 2015, the approval by the Food and Drugs Administration (FDA) of elotuzumab and daratumumab [49] in the treatment of RRMM patients increased the therapeutic armamentarium for MM [50,51]. With these new therapies arose several new treatment strategies, called “algorithm for management of MM” in the publication of Kumar et al. [52]. Nevertheless, MM remains an incurable disease. In fact, these successive sequences of salvage therapies in RRMM patients contribute to the acquisition of additional mutations and genetic abnormalities, DR development, and disease progression [53,54]. The list of new molecules still in clinical trials is really long. These agents are antibody-therapies, PIs, IMiDs, chimeric antigen receptors, and checkpoints inhibitors, and they all act on the immune system of MM [52], highlighting the essential role of the microenvironment in MM progression. One of the crucial features of the microenvironment is hypoxia, which contributes to tumor cell proliferation, cell survival and drug resistance. Hypoxia’s main actors are two transcription factors, HIF1α and HIF2, mediating the hypoxic response [55]. In order to adjust to the hypoxic microenvironment, the transcription factor HIF1α modulates hundreds of genes in tumor cells, allowing proliferation, cell survival, invasion, angiogenesis, and drug resistance, hallmarks of cancer [56].

Multidrug resistance (MDR) is a common problem to all cancers, including MM. In fact, at diagnosis, the MM patient is “MDR-negative”, however, after several successive therapies, MM patients relapse due to MDR phenotype. This MDR is characterized, among others, by the overexpression of ABC transporter proteins, mainly Pgp, MultiDrug Resistance Protein 1 (MRP1), and Breast Cancer Resistance Protein (BCRP) [57]. The detection of Pgp or BCRP are linked to a low response to treatment [57,58]. Resistant clones, possibly derived from side populations (cancer stem cell), have been detected in RRMM patients which overexpress ABC transporter proteins [59,60]. Many agents used in MM treatment are substrates of the Pgp pump, such as PIs, like Btz, and also, carfilzomib [57]. Hence, we set up an MM resistant cell line by using doxorubicin, as this drug induces an overexpression of ABC transporter [61]. IMiDs and PIs constitute the first line therapy in newly diagnosed and also RRMM, and the combination of Vincristine/Adriamycin/Dexamethasone (VAD) is less and less used, due to high toxicity and poor efficacy. The topoisomerase 2 inhibitor, Doxorubicin, is still used in MM treatment as PEGylated liposomal doxorubicin form [62], moreover, recently, a study has been published using the combination VAD in newly diagnosed patients [63].

In this study, we establish a resistant cell line MOLP8/R, selected with doxorubicin, from a sensitive MM cell line MOLP8 displaying a strong expression of the Pgp pump a plasma membrane protein, whose overexpression hijacks drugs from their cell targets [64]. The investigation of the behavior of the resistant clone MOLP8/R to drugs used in frontline therapy in multiple myeloma, IMiDs and PIs, showed that the MOLP8/R cell line is resistant as well to IMiDs (pomalidomide) as well as to PIs (carfilzomib) (Figure 1), drugs both approved by the FDA in MM treatment [2]. The Pgp overexpression results from the pressure with doxorubicin, explaining also the resistance of MOLP8/R to PIs, which are substrates of the pump, but not of IMiDs [65]. The MOLP8 cell line has been isolated from a patient in stage IIIA, which can be already considered as a late stage in the revised classification “International staging system” [66], where MM development is divided in three stages. Stage III, the late stage, is characterized as high risk, due to the cytogenetic abnormalities being able to influence the response to the treatment [67]. Thus, the resistance to pomalidomide of MOLP8/R cell line could be explained by the cytogenetic alteration in MOLP8/R already partly present in the MOLP8 cell line. However, Zagouri et al. demonstrated in a study, done on a cohort of IgD myeloma patients, that they responded identically to new agents as other MM subtypes [68].

On the other hand, another factor can be involved in the drug resistance of MOLP8/R cell line. It has been exhibited that doxorubicin can upregulate HIF1α [69]. In addition, different studies show the promotion of Pgp protein by HIF1α in normoxic condition [56,70]. In fact, the analysis of HIF1α expression in the resistant cell line, MOLP8/R, indicates the upregulation of the protein in normoxia (Figure 3). However, HIF1α overexpression in MOLP8/R cell line does not explain its resistant behavior to IMiDs, especially as it has been exhibited that IMiDs decrease the expression of HIF1α in Bone Marrow (BM) environment in MM [22]. Therefore, we explored the mechanism of action of IMiDs on tumour cells. IMiDs target the E3 ubiquitin ligase cereblon (CRBN) [71], affecting the proteasome activity, as once IMiDs bind to the cereblon complex, this triggers the ubiquitination and degradation of two transcription factors from Ikaros family zinc finger proteins 1 and 3 (IKZF1 and IKZF3) [72], which are involved in MM survival [72]. Since our MOLP8/R cell line is resistant to both therapeutic families, IMiDs and PIs, this highlights also a dysfunction of the proteasome. We confirmed this dysfunction of the proteasome by Western blot (Figure 2), as well as by measuring the proteasome activity (Figure 2), showing lower activity of the proteasome in MOLP8/R cell line. Thus, the deterioration of proteasome activity generates also an accumulation of HIF1α in the resistant clone, as well as the alteration of the pathway, leading to the degradation of HIF1α in normoxia [33]. Generally in normoxia, HIF1α is immediately hydroxylated by propyl hydroxylases (PHD1, 2, 3), allowing the ubiquitination of this complex under the control of VHL, followed by the degradation by the proteasome [73].

The exploration of the “HIF network” [74], in our model, resistant to IMiDs and PIs, exhibits damage in this network (Figure 4). Also, the second pathway, involving factor inhibiting HIF-1 (FIH) in the hydroxylation of HIF1α, the asparagine 803 (Asn 803) [25] is defective in our resistant cell line (Figure 3). The impairment of the HIF “network” [74] achieves the overexpression of HIF1α, HIF-OH, and HIF2 (Figure 3).

The improvement of the frontline therapy in MM treatment results from the effect of drugs on the microenvironment acting on the tumor cells. Among cell types of the immune system constituting the first line of defense of our body, there are the NK cells. In order to escape the immune-surveillance system, tumor cells overcome the immune attack. Thus, the hypoxic microenvironment in MM cells, orchestrated by HIF1 or HIF2 [75], inhibits the NK cell activity on tumor cells in MM [76,77]. In our study, we validated that MOLP8/R cell line is resistant to NK cell lysis at different ratios (Figure 4), and that the resistance to the immune system results from the overexpression of HIF1α observed in the resistance cell line. However, the resistance to NK cell lysis of MOLP8/R cell line could also be based on the resistance to IMiDs. It has been shown that one of the effectiveness of IMiDs in MM treatment is among others due their effect on the immune system by increasing NK cell function [78]. By treating our resistant cell line with a HIF1α inhibitor, cryptotanshinone (CT) (anti-HIF1α), we restored its sensitivity to NK cell lysis to the same level as the MOLP8 cell line (Figure 4), indicating the involvement of HIF1α in the resistance to NK cell lysis in the resistant MOLP8/R. The main role of HIF1α in the drug resistant phenotype of the MOLP8/R cell line is supported by an additional result, obtained by the co-treatment of Btz plus cryptotanshinone 10 µM for 48 h, inducing the recovery of sensitivity of MOLP8/R to Btz with the same IC_50_ as MOLP8 (Figure 6). Thus, to restore the sensitivity of MOLP8/R cell line, HIF1α appears as a potential target, and so we explored, in more detail, the main target genes of HIF1α.

Many lines evidence validated the proposal that the hypoxic microenvironment regulated by HIF1α in tumor cells is responsible for their aggressiveness, their resistance to chemotherapy, and morphologic changes, in order to escape the hostile conditions. All genes involved in these phenomena express a Hypoxia-Response Element (HRE) sequence upstream of their promoter, which is under control of the transcription factor HIF1α. Among these genes, the reference genes regulated by HIF1α are *VEGF*, *GLUT-1*, and *LDH-A* [20]. However, none of these reference genes is upregulated in MOLP8/R cell line in normoxia, in spite of HIF1α overexpression, compared to MOLP8 cell line incubated for 24 h under hypoxic conditions. Thus, all data that we collected to explain drug resistance of MOLP8/R to frontline therapy in MM can be justified by the overexpression of HIF1α.

On the other hand, these results do not explain the sensitivity of our resistant clone to dexamethasone, which has been used for many years in the treatment of MM. Dexamethasone is a glucocorticoid with immunosuppressive properties due to the regulation of glucocorticoid receptor and inhibition of the expression of cytokines [79]. In 2011, Wabnitz et al. demonstrated that dexamethasone also targets the l-Plastin phosphorylation. l-Plastin is an actin-binding protein, and when examining its expression in both cell lines MOLP8 and MOLP8/R, we noticed strong expression of l-Plastin in the resistant clone, by both q-PCR as well as by Western blot (Figures Figure 5b and Figure 6a). l-Plastin is expressed in cells of hematopoietic origin [80], is Ca^2+^-dependent, and is also regulated by the phosphorylation in Ser5 [29,80]. By investigating the level of Phospho l-Plastin expression in MOLP8/R cell line, we confirmed, also, the overexpression of Phospho l-Plastin (Figure 6). According to these results, Phospho l-Plastin appears as a new factor involved in drug resistance in MOLP8/R cell line. The inhibition of Phospho l-Plastin by dexamethasone in the MOLP8/R makes the cell line very sensitive to this drug (Figure 1d). Furthermore, we supported the hypothesis by treating both cell lines with two protein kinase A and C inhibitors of Phospho l-Plastin (Ser5) [36], where we noticed a decrease of Phospho l-Plastin expression in MOLP8/R cell line. However, the decrease of Phospho l-Plastin expression is much higher when the MOLP8/R cells are treated with H89 (Figure 6a). In addition, the co-treatment of MOLP8/R for 48 h with Btz and protein kinase A inhibitor recovers the sensitivity to Btz to the same level as in MOLP8 (Figure 6b). An additional result sustaining the main concern of Phospho l-Plastin in drug resistance of MOLP8/R is the restoration of the sensitivity to pomalidomide, when MOLP8/R cells are co-treated with Dexamethasone at very low concentrations plus pomalidomide (Figure 6c). Yet, so far, we do not know why l-Plastin is overexpressed in the drug resistant cell line. Although the reference genes of HIF1α are not overexpressed, it is know that HIF1α as a transcription factor activates genes with a HRE sequence upstream of their promoters [22]. In silico analysis of the l-Plastin (LCP1) promoter using “EMBOSS Fuzznuc” database revealed two HRE motif (GCGTG) at positions −1527/−1531 and +237/+241 from the transcription start site (TSS). In fact, the study of LCP1 gene on MOLP8 cell line exposed in hypoxic conditions for 24 h shows an overexpression of LCP1 gene, as well as the reference genes (Figure 5). The upregulation of LCP1 gene by HIF1α is also noted, when MOLP8 are treated for 6 h with an inhibitor of HIF1α degradation pathway (Figure 5d).

Albeit the MOLP8/R cell line has been established in vitro, the data of patients support the involvement of l-Plastin in drug resistance in MM. PCL disease (plasma cell leukemia), being an ultimate step of MM, is in fact an aggressive state of relapsed MM patients, where plasma cells pass from BM to the bloodstream, and can thus be considered as a multidrug resistant disease. The analysis of l-Plastin expression in different PCL patients, in parallel to MOLP8, by immunoblot, show a strong overexpression of l-Plastin.

In this work, by setting up a MM resistant cell line MOLP8/R in normoxic condition with Doxorubicin, we establish a drug resistant cell line, resistant to MM frontline therapy, but sensitive to Dexamethasone. Besides the first analysis, showing a strong overexpression of Pgp, our results highlight the overexpression of HIF1α as a potential target involved in drug resistance of MOLP8/R cell line. Yet, our data emphasizes another and new potential target to overcome drug resistance in MM, l-Plastin, whose phosphorylation is inhibited by Dexamethasone. The involvement of l-Plastin as a new marker of drug resistance in MM is confirmed with MM patient samples at a very late stage of the disease. Moreover, to support the importance of Dexamethasone used at low doses in the treatment of MM patients, as described by Kumar et al. in their review from 2017 [52], could be due to its action on Phospho l-Plastin.

In order to validate the involvement of Phospho l-Plastin in DR of MM, MM cells could be isolated from RRMM patients and injected in mice. The treatment of MM animals with dexamethasone at low doses, combined with Phospho l-Plastin inhibitors, would be investigated, in order to verify if inhibition of the phosphorylation of l-Plastin could overcome DR in MM mice. Then, some clinical assays could be realized with RRMM patients treated with a combination of Dexamethasone and l-Plastin phosphorylation inhibitors.

## 4. Materials and Methods

Cell line MOLP8 isolated from a stage III of multiple myeloma patient [81] was obtained from DSMZ (Braunschweig, Germany), grown in RPMI-1640 medium (Lonza, Bornem, Belgium) supplemented with 10% heat-deactivated fetal bovine serum (FBS) (Lonza, Bornem, Belgium) at a density of 4 × 10^5^ cells/mL. Cells were maintained at 5% CO_2_ at 37 °C, and exponentially growing cells were used in all experiments. Natural killer cell line, NK92MI, obtained from ATCC, is maintained in RPMI supplemented with 10% heat-deactivated fetal bovine serum. The renal cell carcinoma CCRCC cell line 786-O, and WT7, which was derived from 786-O by stable transfection with pRC-HAVHL [82], were a gift from Dr. William Kaelin, Jr. (Dana Farber Institute, Harvard Medical School, Boston, MA, USA) [34].

Blood and BM samples from MM patients were collected after agreement and signature of informed consent. MM plasma cells from patients were isolated from samples by using RosetteSep cocktail (StemCell Technologies, Grenoble, France), according to the manufacturer’s instructions.

### 4.1. Chemicals

Doxorubicine (DOX) (Pfizer, Brussel, Belgium); immunomodulatory drugs (IMiDs), as pomalidomide (Celgene, San Diego, CA, USA); proteasome inhibitors (PIs), as bortezomib (Btz) and carfilzomib (Selleckchem, Huissen, The Netherlands); dexamethasone (bioconnect, Huissen, The Netherlands); DFO (deferoxamine mesylate salt) (Sigma, Diegem, Belgium), DMOG (dimethyloxalyl glycine) (Sanbio, Uden, The Netherlands); JNJ (HIF-PHD inhibitor II) (Sanbio, Uden, The Netherlands), cryptotanshinone; and XTT and PMS are from Sigma (Diegem, Belgium). Dihydrochloride (H89) and bisindolylmaleimide-1 (GF 109203C) were both from Millipore (Overijse, Belgium).

### 4.2. Cell Viability (XTT) Assay

Cells (4 × 10^4^) were incubated for 24 h in 96-well flat-bottomed cell culture plates with 100 μL RPMI culture medium. Cells were then treated with a range of dexamethasone, PIs, and IMiDs, alone or in combination with different molecules, for 48 h. Cell viability was determined by addition of 50 µL of 1 mg/mL XTT formazan solution, incubated for 3 h at 37 °C, and the resulting color produced by viable cells was measured on OPTIMA Elisa plate reader (BMG; LabTech, Champigny/Marne, France) at 450 nm and 630 nm, as a reference wavelength.

### 4.3. Protein Extraction

The proteins are extracted from the different cell lines with RIPA buffer (Merck-Millipore, Bruxelles, Belgium), 1× complete protease inhibitor cocktail (Roche, Mannheim, Deutschland), and phosphatase inhibitors (Sigma, Overijse, Belgium). The protein concentrations were determined by Bradford assay (BioRad, Hemel Hempstead Herts HPS2 7DK, UK). Thirty micrograms of protein samples were separated on 12% SDS-polyacrylamide gels, then transferred onto PVDF (Polyvinylidene difluoride) membrane for 1.5 h. The membrane are blocked 3 h with 5% milk of solution in TBST (Tris-buffered saline/tween), and incubated overnight at 4 °C with the primary antibodies. Goat anti-rabbit and goat anti-mouse secondary antibodies (Jackson Immuno Research Laboratories, Sulfolk, UK) were used as appropriate, and detected with the ECL Advance Detection Kit (GE Healthcare, Diegen, Belgium) on Kodak films (Thermofisher, Merelbeke, Belgium).

### 4.4. Antibodies

HIF1α, HIF2, HIF-OH, FIH, GAPDH were obtained from Cell signaling, while PHD1, PHD2, and PHD3 were acquired from Abcam, Pgp from Enzo, actin from Sigma, and l-Plastin from LAB VISION. Ubiquitin antibody was from DAKO.

Phospho-l-plastin antibody was a gift from Dr. Konrad Pazdrak of Biochemistry and Molecular Biology from the Institute for Translational Sciences. VHL antibody was a gift from Dr. Arlot Yannick of Insitut de Genetique & Development de Rennes.

### 4.5. Measure of the Proteasome Activity

We used Amplite™ Fluorimetric Proteasome 20S Activity Assay Kit (Green Fluorescence, Kampenhout, Belgium). The protocol was followed as was recommended.

Briefly, after 1 h incubation at 37 °C and 5% CO_2_, cells are plated in black transparent flat plates 96 wells (Falcon), and 100 μL LLVY-R110 substrate, prepared as indicated in the protocol, was added. Cells are incubated with the substrate for 30 min at 37 °C, 5% CO_2_, in the dark.

The cleaved substrate generates green fluorescent light measured with EL microplate reader (OPTIMA Elisa plate reader (BMG; Isogen Life Science, PW De Meern, The Netherlands), with an excitation wavelength of 490 nm, and an emission wavelength of 525 nm at the time-point 30 min; time-point = 0 min corresponds to an auto-fluorescence test.

### 4.6. Resistance Assay to NK92 Cytotoxicity

Target cells were stained with 5 µM of carboxyfluorescein succinimidyl ester (CFSE) (Thermofisher, Merelbeke, Belgium), and were seeded in 96-well plates at a concentration of 4 × 10^5^ cells/mL. NK-92 were put in contact with tumor cells at different ratios (1 tumor cell per 1, 5, 10, and 30 of NK cells). Cells were incubated together for 4 h at 37 °C, CO_2_ 5%. After incubation, TO-PRO3 Final concentration = 0.3 µM, ThermoFisher Scientific) was added to co-culture just before flow cytometer acquisition (CytoFLEX, Analis, Suarlée, Belgium). Lysis of target cells by NK-92 was evaluated.

### 4.7. RNA Extraction

RNA extractions were performed with EXIQON miCURY^®^ RNA Isolation Kit (EXIQON, Vedbaek, Denmark). Sample concentrations were evaluated with Nanodrop (N60 NPOS 1.4 build 10558).

### 4.8. q-PCR Protocol

Reverse transcription: RT-PCR was done on 200 ng RNA in a 20 μL volume, using the Reverse Transcriptase Core Kit ref RT-RTCK-03 (Eurogentec, Seraing, Belgium).

q-PCR was performed on a VIIA7 machine (Applied Biosystems, Courtaboeuf, France) in MicroAmp Fast 96-well reaction plate (0.1 mL) in a 10 μL reaction volume (Thermofisher, Merelbeke, Belgium). Samples were run in triplicates, and were made of 5 μL Power SYBR Green PCR Master Mix (Applied Biosystems), 4 μL cDNA diluted previously 10 times, and 1 μL primer mix at 2.5 μM.

Samples were run with Fast 96 well ddCT SYBR program: 1 cycle 50 °C 2 min, 95 °C 10 min, followed by 40 cycles of 95 °C 15 s, 60 °C 1 min, and a melting curve.

Primer sequences were HIF2 5′-GCGCTAGACTCCGAGAACAT-3′ and 5′-TGGCCACTTACTACCTGACCCTT-3′, VEGF 5′-GCACCCATGGCAGAAGG-3′ and 5′-CTCGATTGGATGGCAGTAGCT-3′, LDH-A 5′-ATCTTGACCTACGTGGCTTGGA -3′ and 5′-CCATACAGGCACACTGGAATCTC-3′, GUT-1 5′-GATTGGCTCCTTCTCTGTGG-3′ and 5′-TCAAAGGACTTGCCCAGTTT-3′ and l-Plastin forward 5′-TTGGCACCCAACACTCCTA-3′ reverse 5′-CCAGGGCTTTGTTTATCCAG-3′.

### 4.9. Data Are Calculated as a Fold Change Compared to Reference Sample

The statistical analysis was done with Excel software by using *t* test for the validation. * means *p* < 0.05, ** represents *p* < 0.01, *** *p* < 0.001, **** *p* < 0.0001 and ***** *p* < 0.00001.

## Abbreviations

MM	multiple myeloma
IMiDs	immunomodulatory drugs
PIs	proteasome inhibitors
HIF1α	hypoxia-inducible factor 1-alpha
ABC	transporter ATP-binding cassette transporter
Pgp	P-glycoprotein
MRP1	multidrug resistance-associated protein 1
BCRP	breast cancer resistance protein
MDR	multidrug resistance
HRE	hypoxia-response element
FDA	Food and Drug Administration
PHD	prolyl hydroxylase
FIH	factor-inhibiting HIF1α
NK	natural killer
VEGF	vascular endothelial growth factor
GLUT1	glucose transporter 1
LDH-A	lactate dehydrogenase A
PKA	protein kinase A
